# Stimulatory, but not anxiogenic, doses of caffeine act centrally to activate interscapular brown adipose tissue thermogenesis in anesthetized male rats

**DOI:** 10.1038/s41598-020-80505-9

**Published:** 2021-01-08

**Authors:** L. Van Schaik, C. Kettle, R. Green, W. Sievers, M. W. Hale, H. R. Irving, D. R. Whelan, J. A. Rathner

**Affiliations:** 1 Department of Pharmacy and Biomedical Sciences, La Trobe Institute of Molecular Science, La Trobe University, Bendigo, VIC Australia; 2 Department of Psychology, School of Psychology and Public Health, La Trobe University, Bundoora, VIC Australia; 3 Department of Physiology, School of Biomedical Sciences, The University of Melbourne, Parkville, VIC Australia

**Keywords:** Neuroscience, Physiology

## Abstract

The role of central orexin in the sympathetic control of interscapular brown adipose tissue (iBAT) thermogenesis has been established in rodents. Stimulatory doses of caffeine activate orexin positive neurons in the lateral hypothalamus, a region of the brain implicated in stimulating BAT thermogenesis. This study tests the hypothesis that central administration of caffeine is sufficient to activate BAT. Low doses of caffeine administered either systemically (intravenous [IV]; 10 mg/kg) and centrally (intracerebroventricular [ICV]; 5–10 μg) increases BAT thermogenesis, in anaesthetised (1.5 g/kg urethane, IV) free breathing male rats. Cardiovascular function was monitored via an indwelling intra-arterial cannula and exhibited no response to the caffeine. Core temperature did not significantly differ after administration of caffeine via either route of administration. Caffeine administered both IV and ICV increased neuronal activity, as measured by c-Fos-immunoreactivity within subregions of the hypothalamic area, previously implicated in regulating BAT thermogenesis. Significantly, there appears to be no neural anxiety response to the low dose of caffeine as indicated by no change in activity in the basolateral amygdala. Having measured the physiological correlate of thermogenesis (heat production) we have not measured indirect molecular correlates of BAT activation. Nevertheless, our results demonstrate that caffeine, at stimulatory doses, acting via the central nervous system can increase thermogenesis, without adverse cardio-dynamic impact.

## Introduction

Energy homeostasis plays a crucial role in maintaining the health of animals, and is specifically regulated by the central nervous system, which receives and integrates peripheral signals of energy status and modulates energy expenditure and food intake^1^. The autonomic nervous system is involved in the regulation and innervation of metabolic organs and physiologically responds to both exogenous and endogenous stimuli^2,3^. Within the autonomic nervous system, the sympathetic nervous system has a regulatory role in thermogenesis and energy expenditure^4^. When activated, the sympathetic nervous system increases energy expenditure, particularly resting energy expenditure^5^. Brown adipose tissue (BAT) is known to be innervated by the sympathetic nervous system and is a regulator of energy homeostasis^5^. Adipose tissue found in mammals is categorised into three types of adipocytes white, brown, and beige/brite and two types of tissue white adipose tissue (WAT) and BAT^6,7^. BAT is a specialized tissue that dissipates energy in the form of heat through non-shivering thermogenesis^8,9^, WAT stores energy and is an important endocrine organ^9^. Beige/brite adipocytes are “brown-like” cells within white adipose depots, and have a gene expression pattern distinct from either white or brown fat^10^. The thermogenic ability of BAT is due to uncoupling protein 1 (UCP1), which facilitates the futile cycle in the mitochondria of brown adipocytes^11^. The discovery that BAT has a significant role in human energy homeostasis has generated considerable interest in it as a viable target for pharmacologically increasing metabolism^12^. In rodents BAT is sympathetically regulated through β-3 adrenoceptors^13^, however in humans the β-adrenoceptor primarily involved in its activation is less clear^14–16^. Electrical stimulation of the nerves innervating BAT or peripheral administration of a β adrenoceptor agonist in mice, increases downstream signalling of the adrenergic receptors within two hours, and is accompanied by lipolysis^17^. The sympathetic nervous system also acts on the cardiovascular system, but in humans, there appears to be evidence of β3 receptors in myocytes and cardiac tissues, however at much lower levels than other beta adrenergic receptors^18^.

The neural pathway that controls the regulation of BAT thermogenesis has been established in rodents^5^. Several hypothalamic nuclei are associated with the regulation of BAT thermogenesis^4^. Interestingly, control of BAT appears to follow a neural pathway which is exclusive and distinct from that which controls the cardiovascular system^19^. While there is overlap^20^, the neural pathway controlling the cardiovascular system appears to be different to that which controls thermoregulatory reflexes^21^. The rostral raphe pallidus neurons regulate sympathetic outflow to BAT, whereas the rostral ventrolateral medulla mediates cardiac sympathetic discharge^22^. Additionally, there is strong evidence that indicates the premotor neurons in the central nervous system that regulate BAT are in fact specific and are not involved in regulating cutaneous vasomotor activity^21^.

Caffeine is a psychoactive drug which acts in humans to stimulate thermogenesis and the breakdown of lipids^23–25^. Historically, research investigating the effects of caffeine have focused on its peripheral effects^24^. However, caffeine’s effect on arousal is central in origin^26^, raising the possibility that caffeine evoked thermogenesis may have a central component. In rat studies, low (but stimulatory) doses of caffeine have previously activated orexinergic neurons in the dorsomedial hypothalamus (DMH) and the perifornical areas of the lateral hypothalamus (PeF/LH)^27,28^. These are regions of the hypothalamus that have been shown to regulate sympathetic nerve activity to iBAT, leading to thermogenesis^29^.

Orexins (hypocretins), are synthesised in neurons located in the LH, and PeF/LH^30^. Orexin receptors and axon terminals containing orexin are located in the rostral medullary raphé (rRPa)^29^, which is a site of sympathetic premotor neurons involved in thermoregulation^31^. Orexins have a recognized role in the management of body temperature and controlling heart rate, energy expenditure and BAT thermogenesis^32–35^. Direct application of orexin into the rRPa activates sympathetic nerve activity to iBAT^36^.

This evidence suggests a central neural pathway for thermogenesis, specifically for promoting energy wasting. Caffeine may activate this pathway, which could potentially explain caffeine’s increase in energy expenditure, without having adverse hypertensive effects and tachyarrhythmia.

The amygdala is an important component of anxiety-related neuronal circuitry^37^. Although caffeine is consumed as a stimulant, promoting arousal, high doses of caffeine cause anxiety^38–40^. Anxiety is associated with increased muscle tension, blood pressure, respiration and tachycardia^41^. Anxiety is thought to be regulated by the amygdala, and high doses of caffeine, sufficient to induce anxiety like behaviour in rats, have been shown to activate neurons in this nucleus^42^.

Here we aim to test the hypothesis that a central administration of stimulatory dose of caffeine is sufficient to activate iBAT thermogenesis. We test the efficacy of caffeine delivered either systemically (intravenous, IV administration) and centrally (intracerebroventricular, ICV administration) in eliciting increased iBAT and core temperature in rats. Since the physiological end point of thermogenesis (excess heat production) is determined, measures of molecular correlates of BAT thermogenesis, such as UCP1 expression or downstream adrenergic signalling were not conducted. Findings of the present study demonstrate that caffeine, at stimulatory, but non anxiogenic doses, acts through the central nervous system to increase thermogenesis, without adverse impact on mean arterial pressure and heart rate.

## Results

### Caffeine increases iBAT temperature

**Figure 1:**
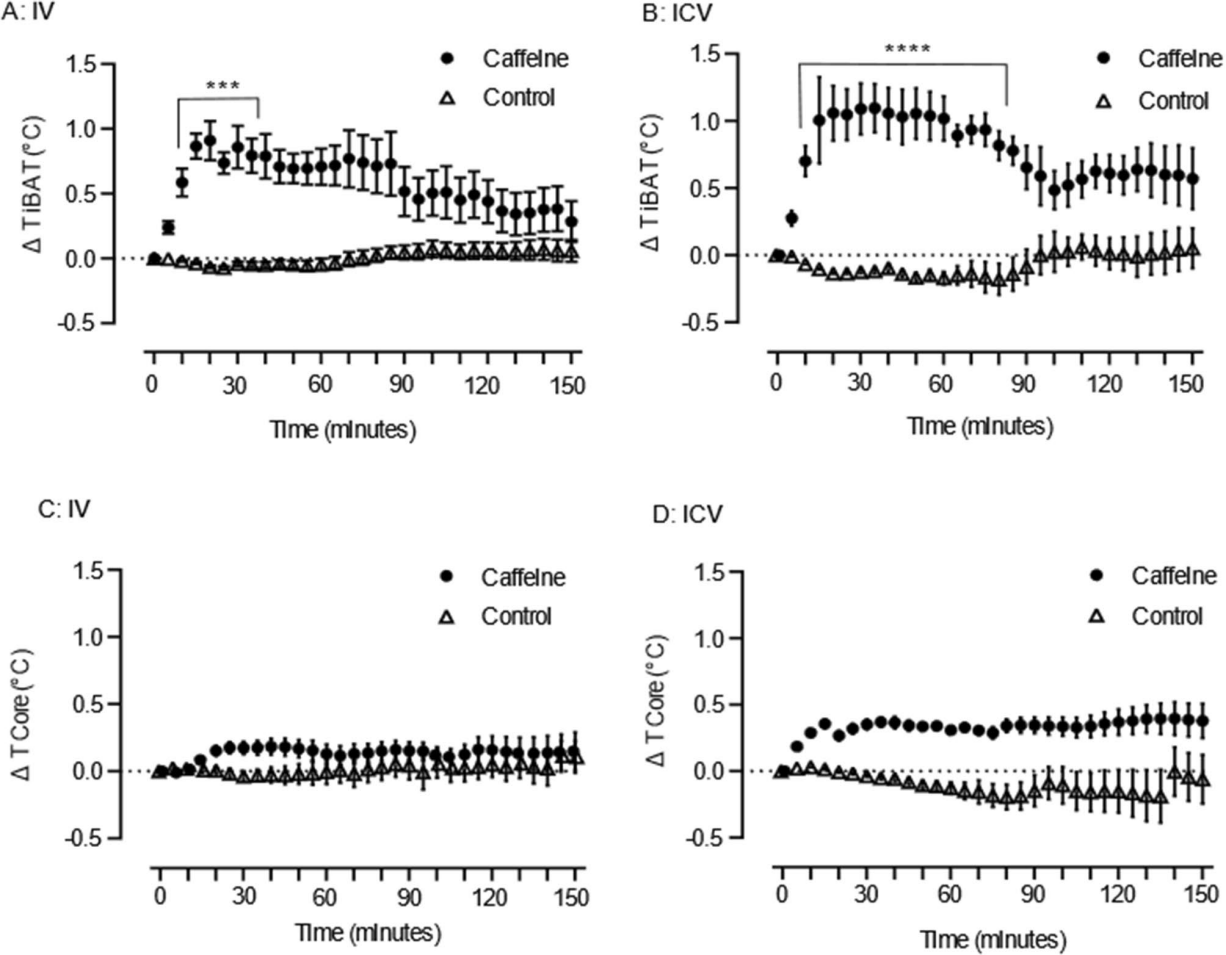
Changes in temperature (ΔT) of core and interscapular brown adipose tissue (iBAT) in rats following an administration (time = zero) of caffeine or vehicle alone to 150 min post treatment. Temperature of iBAT (**A**) IV administration † and (**B**) ICV administration. Core temperature (**C**) IV administration † and (**D**) ICV administration. Filled circle = Caffeine, open triangle = Control. Error bars represent S.E.M., *n* = 8 per intervention, *represents interaction effect (***p < 0.001, ****p < 0.0001), † raw data shown in figure, but data were transformed prior to analysis (see “Materials and methods” section).

To test if caffeine increased iBAT temperature, we used a parallel experimental design administering caffeine or saline (vehicle) treatment to male Sprague Dawley rats peripherally by IV or centrally by ICV delivery. The doses of caffeine both IV and ICV were based on previous studies measuring the stimulant, but non-anxiogenic doses of caffeine, where the dose did not promote any behavioural impairment^43–45^. These doses of caffeine are typically consistent with normal coffee consumption in humans. Core and iBAT temperature were measured to assess the change in temperature following the intervention, and therefore degree of thermogenesis. iBAT and core temperature were recorded via placement of thermocouples rectally (core) or underneath iBAT and analyses were conducted separately for IV and ICV administration. Stable temperature was maintained for 30 min prior to drug administration. Baseline core and iBAT temperature are summarised in Table 1 showing no significant difference in base line conditions for any of the physiological measures (p > 0.05, t-tests). For IV administration a significant interaction effect (F_(30, 420)_ = 3.5, *p* < 0.0001, Fig. 1A) identified that caffeine (10 mg/kg) increased iBAT temperature between 10 and 35 min after administration with no change in temperature for the vehicle alone group. The peak increase in temperature for the caffeine group (1.1 °C ± 0.2 °C) was 25 min after administration. Similarly, ICV administration of caffeine (5–10 µg) saw a significant interaction effect (F_(30, 420)_ = 3.5, *p* < 0.0001, Fig. 1B), with no change in temperature for the vehicle alone group. Increases in iBAT temperature (0.7 ± 0.1 °C) were at 10 min with a peak rise in temperature (1.2 ± 0.2 °C) at 30 min, this was sustained until 85 min (0.9 ± 0.2 °C). Neither IV nor ICV caffeine administration evoked changes in core body temperature (IV: F_(30, 420)_ = 0.8_,_ *p* = 0.6; Fig. 1C ICV: F_(30, 330)_ = 0.9_,_ *p* = 0.5; Fig. 1D).

**Table 1:** Average baseline physiological measures prior to drug administration: statistical testing indicates no difference in base line conditions prior to interventions.

	HR (BPM)		MAP (mmHg)		Core temperature (°C)		Intra-iBAT temperature (°C)	
IV control	390 ± 10.7	0.5*	136.46 ± 3.5	0.5*	37.1 ± 0.1	0.6*	34.2 ± 0.1	0.1*
IV caffeine	393 ± 9.7		137.68 ± 4.5		37.3 ± 0.1		34.5 ± 0.2	
ICV control	397 ± 9.5	0.8*	127.48 ± 6.5	0.2*	37.3 ± 0.2	0.4*	34.6 ± 0.3	0.1*
ICV caffeine	396 ± 9.7		123.45 ± 6.2		37.2 ± 0.3		34.8 ± 0.2	

*Indicates p-value calculated using an unpaired t-Test, Welch’s correction, *n* = 8 per intervention.

### Dose of caffeine used does not significantly change heart rate or mean arterial pressure

**Figure 2:**
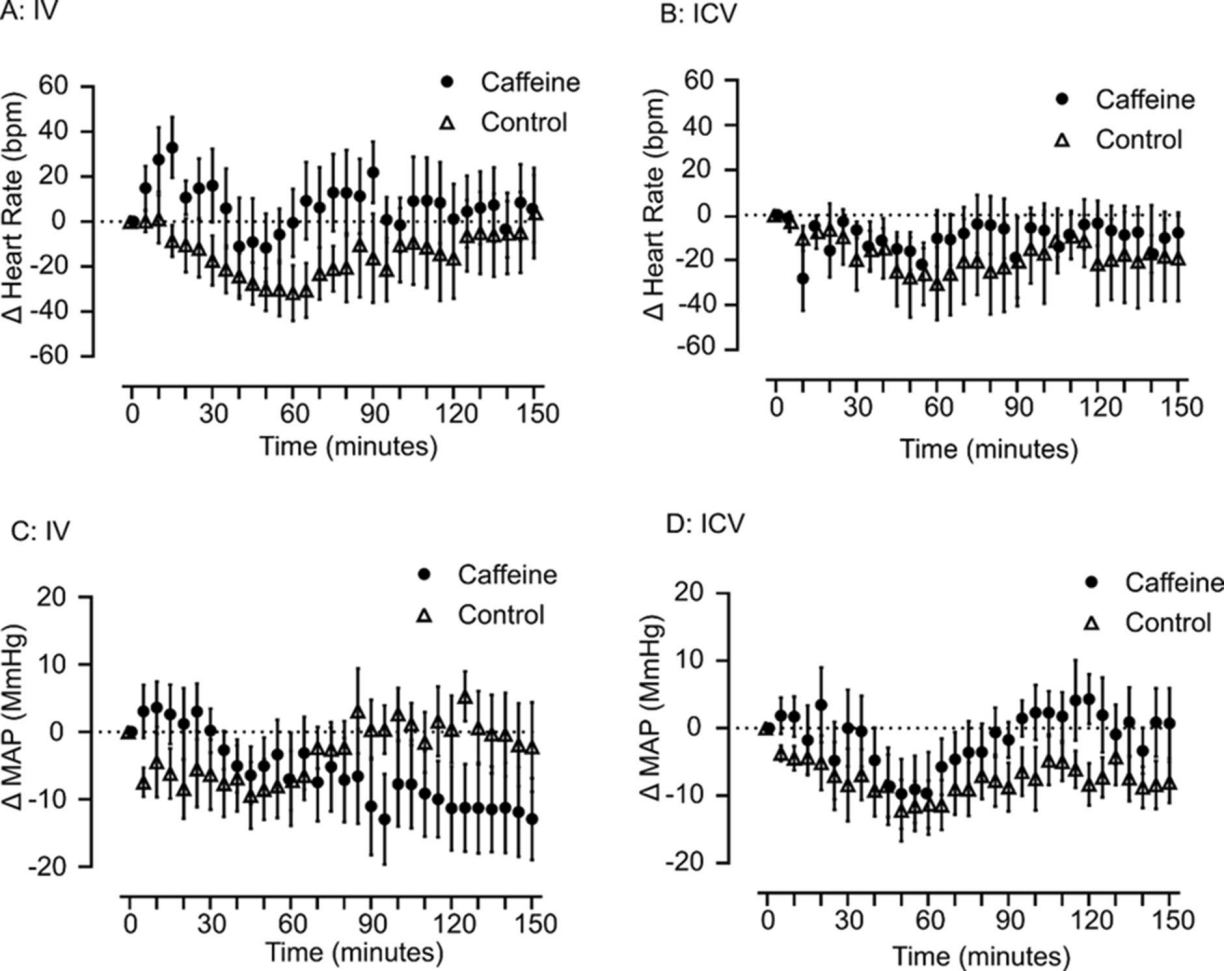
Changes in heart rate and mean arterial pressure in rats following an administration of caffeine. Changes in beats per minute of heart rate from baseline to 150 min post treatment of caffeine or vehicle (**A**) IV †; (**B**) ICV †. Changes in mean arterial pressure from baseline to 150 min post treatment (**C**) IV †; (**D**) ICV †. Filled circle = Caffeine, open triangle = Control. Error bars represent S.E.M., *n* = 8 per intervention, † raw data shown in figure, but data were transformed prior to analysis (see “Materials and methods” section).

To test whether the dose of caffeine affected cardiovascular responses, in response to either route of administration of caffeine, heart rate and mean arterial pressure were measured via intra-arterial catheter. Analysis was conducted separately for IV and ICV administration. Baseline heart rate and mean arterial pressure data are summarised in Table 1*.* Temperature and cardiovascular measures remained stable for 30 min prior to drug administration and there was no observed difference in baseline conditions prior to intervention. Neither IV or ICV administration of caffeine changed heart rate compared to vehicle alone over time (interaction effects: IV: F_(30, 420)_ = 1.0_,_ *p* = 0.4, Fig. 2A; ICV: F_(30, 330)_ = 0.3_,_ *p* = 0.9, Fig. 2B). Systemic (IV: Fig. 2C) or central (ICV: Fig. 2D) administration of caffeine had no effect on mean arterial pressure compared with vehicle alone (interaction effects: IV: F_(30, 420)_ = 1.3_,_ *p* = 0.1; ICV: F_(30, 330)_ = 0.3_,_ *p* = 0.9).

### Caffeine administration increases activity of hypothalamic nuclei: PaAP, MPA, LH, PeF/LH, DMH, VMH, CM and PVP, but not BLA

**Figure 3:**
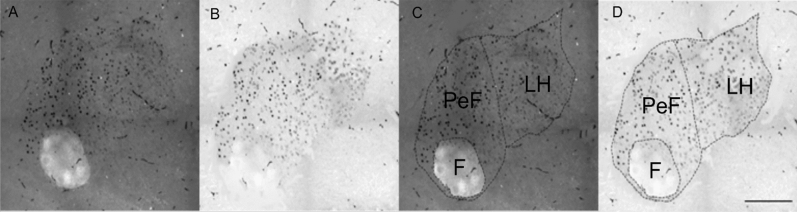
Photomicrographs illustrating the use of brightfield and darkfield microscopy for precise demarcation of anatomical boundaries in low background c-Fos-ir sections. (**A**) Darkfield images were utilized to reveal the detailed neuroanatomy by the contrast between myelinated tracts/areas and grey matter. (**B**) Brightfield photomicrographs of identical sections revealed c-Fos-ir nuclei; (**C**) the darkfield photomicrographs were used to precisely outline regions of interest (ROI). (**D**) The ROI was transferred to the brightfield micrograph for automated cell counting. *F* fornix, *PeF* peri-fornix, *LH* lateral hypothalamus.

**Figure 4:**
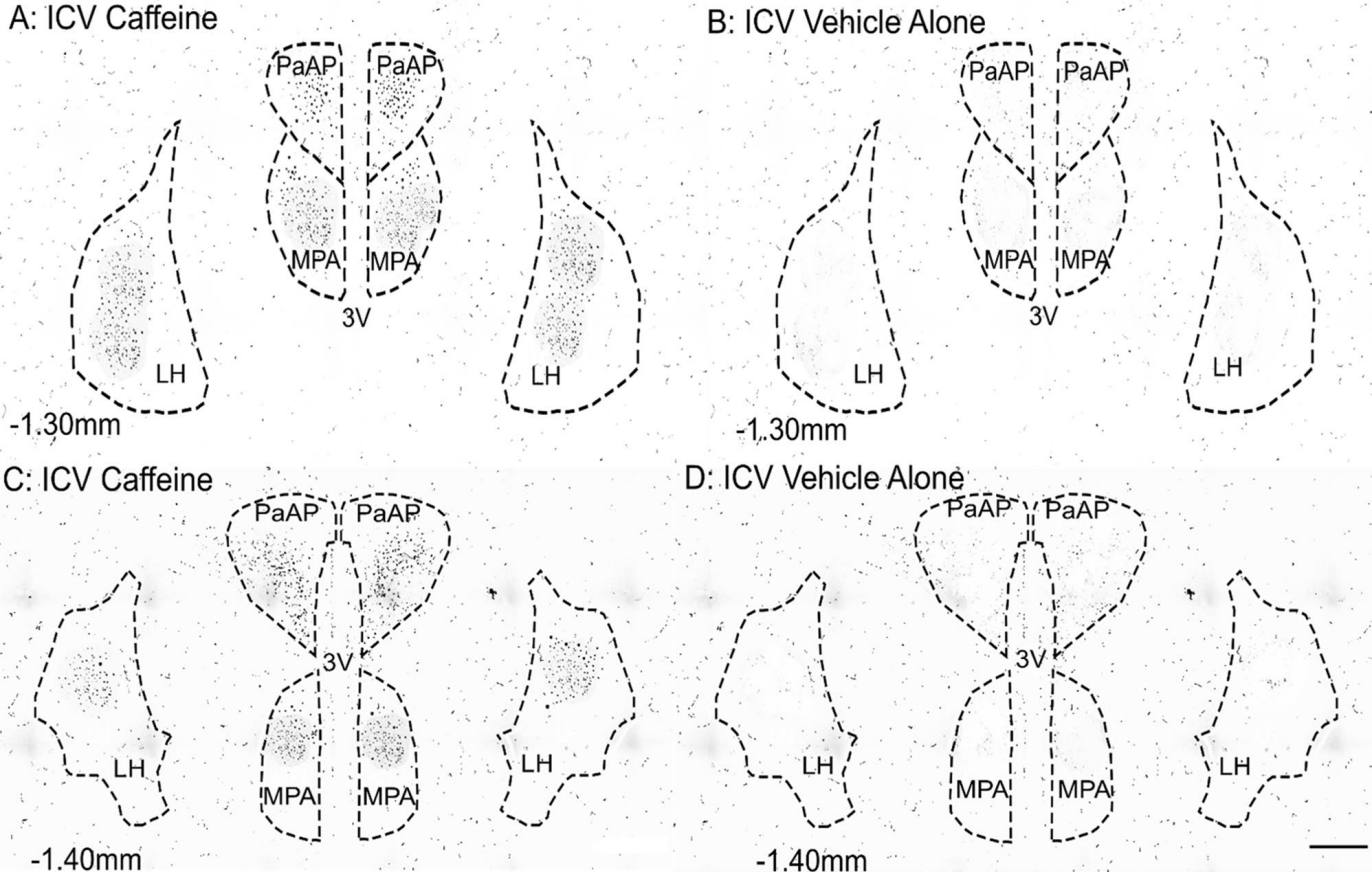
Photomicrographs illustrating the rostrocaudal levels of the paraventricular hypothalamic nucleus anterior parvo (PaAP), medial preoptic area (MPA), lateral hypothalamus (LH) delineation of regions by immunostaining of cFos ir (dark speckled puncta on image): (**A**) − 1.30 mm bregma of caffeine treated rats; (**B**) − 1.30 mm bregma of vehicle treated rats; (**C**) represent -1.40 mm of caffeine treated rats; (**D**) − 1.40 mm bregma of vehicle treated rats. Dashed lines represent regions of interest. *3 V* 3rd ventricle. Scale bar, 200 µm.

**Figure 5:**
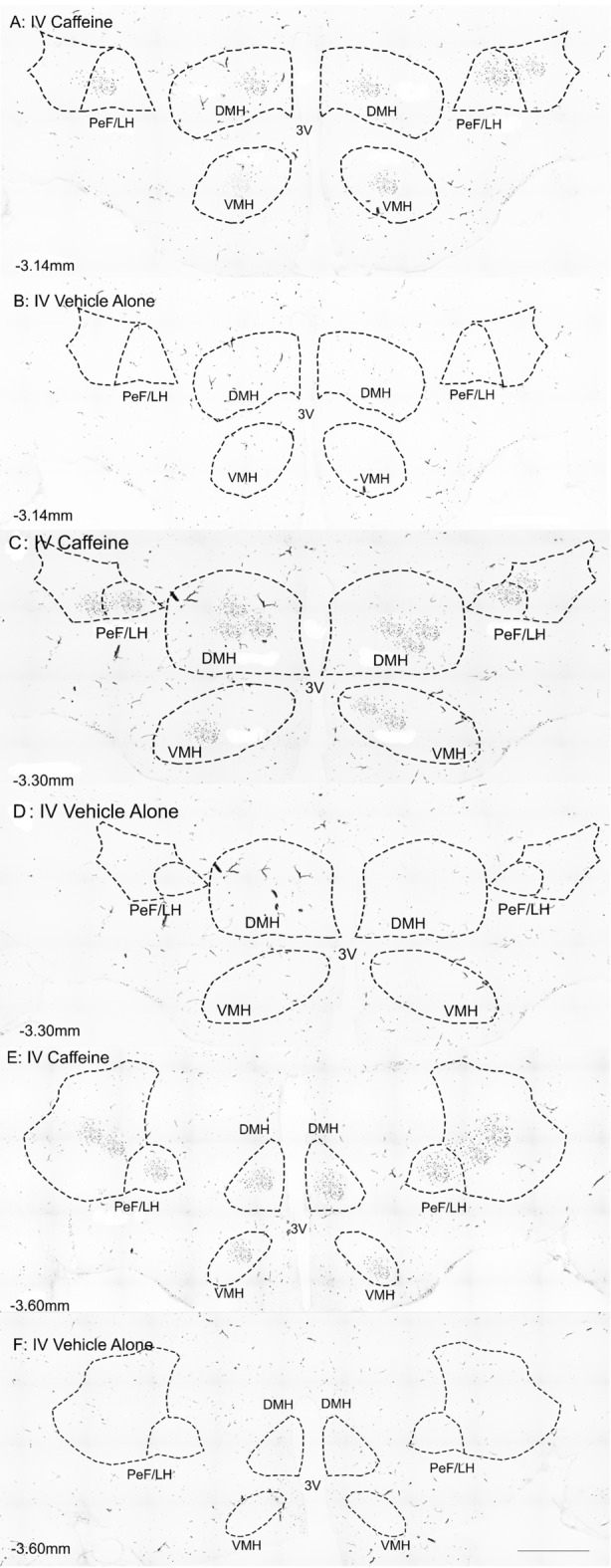
Photomicrographs illustrating the rostrocaudal levels of caffeine treated rats. Areas include the dorsomedial hypothalamus (DMH, ventromedial hypothalamus (VMH), and peri-fornical area of the lateral hypothalamus (PeF/LH) selected for analysis, and delineation of regions by immunostaining of c-Fos ir (dark speckled puncta on image): (**A**) represents − 3.14 mm bregma of caffeine treated rats; (**B**) represents − 3.14 mm bregma of vehicle treated rats; (**C**) represents − 3.30 mm bregma of caffeine treated rats; (**D**) represents − 3.30 mm bregma of vehicle treated rats; (**E**) represents − 3.60 mm bregma of caffeine treated rats; (**F**) represents − 3.60 mm bregma of vehicle. Dashed lines represent regions of interest. *3 V* 3rd ventricle. Scale bar, 200 µm.

To test whether the dose of caffeine activated central hypothalamic circuitry, activation of hypothalamic nuclei was assessed via immunohistochemical analysis of the number c-Fos-immunoreactive (c-Fos-ir) cells. Analysis was performed using an automated counting method that was independently tested with an assessor blinded to sample type to show very good reliability (ICC_3,1_ = 0.99). Every fourth coronal 30 µm section of the hypothalamic region of each rat brain was probed for c-Fos-ir. Different areas of the hypothalamic regions were identified as described in Paxinos and Watson^46^. In order to demarcate the boundaries of different regions, darkfield and brightfield photomicrographs were taken and reconstructed via automatic stitching using Olympus CellSens software (Fig. 3). Two rostrocaudal levels (− 1.30 mm and − 1.40 mm from bregma) containing the basolateral amygdala (BLA), the paraventricular hypothalamic nucleus (PaAP), the medial preoptic area (MPA), and the LH of each rat were analysed as shown in Fig. 4 for ICV administration. Three further rostrocaudal levels (− 3.14 mm, − 3.30 mm, and − 3.60 mm from bregma) of each rat were analysed. These levels included the PeF/LH, the DMH, the ventromedial hypothalamus (VMH), the paraventricular thalamic nucleus posterior (PVP), and the central medial thalamus (CM), as shown in Fig. 5.

**Figure 6:**
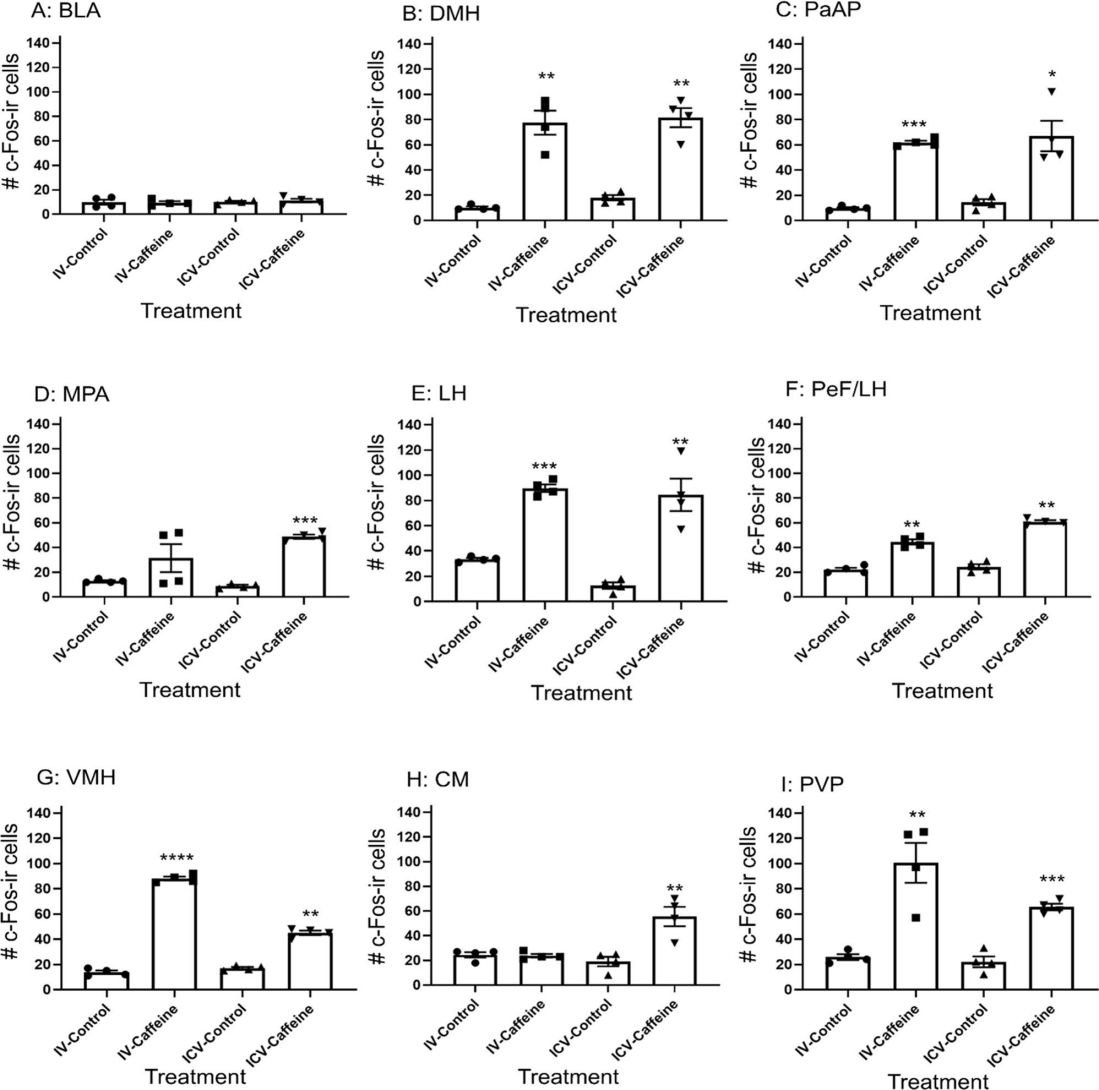
The number of c-Fos-ir neurons in various regions of interest in the hypothalamus following administration of caffeine or vehicle alone via IV or ICV. (**A**) BLA, (**B**) DMH, (**C**) PaAP, (**D**) MPA, (**E**) LH, (**F**) PeF/LH, (**G**) VMH, (**H**) CM, (**I**) PVP. Graphs illustrate the mean difference in number of c-Fos-ir neurons following administration of caffeine or control. Error bars represent S.E.M., *n* = 4, represents caffeine effect *p < 0.05; **p < 0.01; ***p < 0.001; ****p < 0.0001. Analysis conducted separately for IV and ICV administration. *BLA* basolateral amygdala, *PaAP* paraventricular hypothalamic nucleus, *MPA* medial preoptic area, *VMH* ventromedial hypothalamus, *CM* central medial thalamus, *PVP* paraventricular thalamic nucleus posterior.

Intravenous administration of caffeine showed a significant interaction effect on c-Fos-ir activity (F_(8, 54)_ = 266.6, p < 0.0001). Post hoc analysis revealed caffeine treatment had no effect on c-Fos-ir activity in the BLA and CM, whereas for all other nuclei caffeine treatment increased c-Fos-ir (Fig. 6). Administration of caffeine ICV showed a significant interaction effect on c-Fos-ir activity (F_(8, 54)_ = 121.1, p < 0.0001). Post hoc analysis revealed treatment of caffeine ICV had no effect on c-Fos-ir activity in the BLA, whereas c-Fos-ir increased in all other nuclei following caffeine administration (Fig. 6).

## Discussion

This study shows that low doses of caffeine, typically doses consistent with normal coffee consumption in humans, are sufficient to increase BAT thermogenesis. There has been some suggestion in the literature that caffeine’s thermogenic effect may be due to its peripheral actions^47^. Here we show that thermogenesis was evoked by both central and systemic administration. Furthermore, at the doses used in this study, BAT thermogenesis is evoked without increases in either heart rate or mean arterial pressure. This is perhaps the first demonstration of meaningful thermogenesis without cardio-dynamic effects. High doses of caffeine are reported to have anxiogenic effects^38–40^. At the doses used in this study, no increased neuronal activation (as measured by c-Fos-ir) was observed in the basolateral amygdala. Together with the absence of cardio-dynamic response, these data strongly suggest that the doses of caffeine used in this study have not evoked an anxiety response.

In this study we measured evoked thermogenesis as a change in iBAT temperature, with no change in core temperature. It has been previously reported that cooling urethane anaesthetized rats increases iBAT temperature without changes in core temperature, due to increased sympathetic nerve drive to iBAT^48^. Previous studies have shown that increased sympathetic nerve drive to fat increases UCP1 expression^49^. Furthermore, it has also been reported that exogenous caffeine given systemically in mice increases UCP1 expression in BAT^50^. These observations are consistent with our conclusion of caffeine evoked BAT thermogenesis.

Immunohistochemical c-Fos-labeling revealed that regions of the hypothalamus that have previously been implicated in energy homeostasis and, more specifically BAT thermogenesis, are activated as a consequence of both systemic and central administration of caffeine. This is consistent with the hypothesis that it is caffeine’s central actions that leads to its thermogenic response.

Experiments here were performed in urethane anesthetized animals, similar to other studies^23,48^ which have shown thermoregulatory, febrile and evoked BAT thermogenesis due to increased sympathetic nerve drive. The advantage of anesthetized preparations allows for very controlled conditions. Here we were able to observe very consistent and stable baseline conditions prior to intervention. Previous reports have shown that caffeine stimulates increased activity and feeding^51^ which would confound measurements of thermogenesis. As a result, we have eliminated feeding induced thermogenesis. A possible future study expanding from the results obtained in this paper could be carried out on awake animals.

### Physiology/pharmacology of caffeine

The increase in iBAT temperature following ICV administration suggests activation of central circuits. The relative contribution of systemic versus central effect of IV administration of caffeine on BAT thermogenesis remain to be determined. Systemic effects of caffeine at doses much higher than the dose used in this present study have been shown to inhibit the action of cyclic AMP (cAMP) phosphodiesterase^52^, this increases cellular cAMP, which could prolong the effects of sympathetic activation. It is widely acknowledged that caffeine promotes the release of epinephrine, from the adrenal medulla^53,54^, which may have also contributed to caffeine evoked increase in iBAT temperature. Furthermore, caffeine may contribute to the activation of BAT thermogenesis, at least partly, via the mobilization of fuel substrates through the stimulation of lipolysis on white adipose tissue^55^. This study showed that c-Fos-ir is increased in the PeF/LH, LH and the DMH following doses of caffeine administered either by IV or ICV. These are areas known to that contain orexinergic neurons^28,29,56,57^. Activation of neurons in the DMH is sufficient^5^ and necessary to increase sympathetic nerve activity^21^ to iBAT and activate BAT thermogenesis. Activation of neurons within the LH activate iBAT sympathetic nerve activity and thermogenesis^57^. Additionally, orexinergic neurons innervate the PVP^58^, where we show caffeine evoked activation of neuron activity.

The amygdala is an important component of anxiety-related neuronal circuitry^37^. Doses of caffeine used in this study (ICV: 5–10 µg/kg; IV: 10 mg/kg) did not produce a significant increase in c-Fos-ir in the BLA (Fig. 6a). This contrasts with research showing caffeine at much higher doses (50 mg/kg/I.P) significantly increasing c-Fos-ir in the BLA^45^ and increased anxiety like behaviour in male rats exposed to an elevated plus maze^59^.

This study demonstrates that a systemic or central administration of caffeine into the lateral ventricle activates the PaAP. The paraventricular nucleus (PVN) is an area of the brain that contains parvocellular neurosecretory neurons where oxytocin cells have been shown to be involved in energy metabolism^60^. Parvocellular vasopressin cells previously shown to be involved in BAT thermogenesis project centrally to a number of hypothalamic regions, including the DMH^61^. Previous research that has stimulated the PVN have concluded that this stimulation activates iBAT thermogenesis in rats^62^. Caffeine inhibits hypothalamic adenosine A1A receptors, which are expressed on oxytocin cells within the PVN neurons, this negatively regulates energy balance in obese mouse models^63^.

### LH and BAT thermogenesis

Analysis of c-Fos-ir data revealed that administration of caffeine both systemically and centrally increased c-Fos expression within the LH. It has been previously shown that disinhibition of LH neurons stimulated a significant increase in nerve activity to BAT^57^, accompanied by an increase in iBAT temperature, increases in heart rate (+ 88 bpm) and mean arterial pressure (+ 11 mm Hg)^57^. The observed increases in mean arterial pressure and heart rate stand in contrast with the present study, where we have observed no significant cardio-dynamic effect. The orexin activation of BAT thermogenesis in a previous study increased BAT temperature by approximately 1.9 °C^16^, which is slightly higher than the BAT temperature rise observed in this study. This activation of BAT thermogenesis in the previous study was coupled with significant increases in core temperature and heart rate, but no change in mean arterial pressure^29^. The approximate 0.7 °C difference in temperature rise seen between this prior study^29^ and our study suggest that the earlier study evoked higher overall levels of sympathetic nerve activation than observed here. This further highlights the significance of our findings where the doses of caffeine used, evoked increases in BAT thermogenesis without increases in either heart rate or mean arterial pressure. A finding perhaps reported for the first time.

### Caffeine as an adenosine receptor antagonist

Caffeine works centrally to promote arousal through antagonism of the adenosine A1A receptor^28^, which releases orexinergic neurons from disinhibition^28^. This suggests that central effects of caffeine on BAT thermogenesis are through its antagonistic action on the A1A receptor. This observation is harmonious with a role of orexin/hypocretin as a key neuropeptide in the regulation of energy homeostasis and the sleep–wake cycle^27^. Recently it has been demonstrated that there is an increase in both brain adenosine and A1AR in the hypothalamus associated with the development of obesity in mice^63^. Overexpression of A1AR in the PVN is sufficient to induce obesity and suppress BAT thermogenesis^63^. Finally, inhibition of A1AR with systemic caffeine has been shown to reduce body weight, increase BAT thermogenesis and increase oxygen consumption in high fat diet-induced obese rats^63^.

Systemically, caffeine can activate thermogenesis^64^, through its effects on the adenosine A2A receptor^65^, as the A2A receptor is abundantly expressed on BAT^66^. Furthermore, adenosine A2A and A2B agonists increase lipolysis and BAT thermogenesis^66^, whereas adenosine A1 antagonism increased cAMP abundance and oxygen consumption. Expression of A2A receptors is increased in cold-exposed mice as well as in brown adipocytes in response to norepinephrine or cAMP^66^.

As caffeine is a non-specific adenosine antagonist it may influence multiple peripheral mechanisms that act paradoxically upon adipose tissue and thermogenesis^66^. From the findings of this study, it seems likely that systemically administered caffeine is crossing the blood brain barrier and acting centrally to elicit thermogenesis. The significance of our finding is that we have observed that either a systemic or central administration of caffeine at the doses used increases iBAT temperature, without a cardio-dynamic effect. As such there remains potential to investigate the effect a combination therapy of caffeine and a selective adenosine A2A or A2B agonist on thermogenesis. Such a combination therapy may increase the efficacy of caffeine.

## Conclusion

Here we have measured the physiological outcome of thermogenesis, which is heat generation (i.e. temperature change). We observed increases in interscapular temperature without changes in core temperature, most likely explained by BAT thermogenesis. Molecular markers are noted to be useful correlates of BAT activation; hence a possible limitation of this study may be a lack of molecular analysis of UCP1 or downstream signaling of adrenergic receptors. In conclusion, we show that a stimulant, but low dose of caffeine administered systemically and centrally increases iBAT temperature (Fig. 1a,b) and also increases c-Fos-ir in nuclei within the hypothalamus (Fig. 6) that has been implicated with driving BAT thermogenesis. This is without having significant effects on core temperature (Fig. 1c,d), heart rate and mean arterial pressure (Fig. 2). There is still a need to determine the direct effects of caffeine in each of these brain regions (Fig. 6). However, our results demonstrate that caffeine, at stimulatory doses acts via the central nervous system to increase BAT thermogenesis.

## Materials and methods

### Animals

Using G-Power, a power analysis was conducted based on data presented in Yoshioka et al.^67^, showing a 0.2 ± 0.1 °C increase in iBAT temperature after 40 mg/kg intraperitoneal caffeine injection, with an effect size of 2. Based on results from the power analysis we required a minimum of 6 animals per treatment per group to claim a statistically significant change in BAT temperature.

All studies were conducted on male Sprague Dawley rats (*N* = 32, 300–400 g, 8 animals per treatment), that were housed as pairs in a temperature-controlled environment (22° ± 1 °C) with a 12:12 h light and dark cycle from 7pm–7am and fed standard chow. Only male rats were used as estrogen and therefore, the estrous cycle effects BAT thermogenesis^68^. Before each experiment the animals were adapted for 5–7 days to room and cage conditions and had access to water and food ad libitum. Testing began at the same time for each experiment. Animals were randomly assigned into two treatment and administration groups (caffeine ICV/IV or control ICV/IV) with 8 animals in each group. All procedures were conducted in accordance with the NHMRC Australian code for the care and use of animals for scientific purposes (8th edition, 2013) and approved by the La Trobe University Animal Ethics Committee (AEC 15-46).

### Surgical procedure

Anaesthetic induction was completed via intraperitoneal injection of pentobarbitone (30–50 mg/kg, diluted lethabarb, Virbac Animal Health). Surgical tracheotomy was completed with a 16-gauge venous cannula inserted into the trachea. Once cannulation of the jugular was successfully performed anaesthetic depth was maintained via IV administration of urethane (1–1.5 mg/kg). Intra-arterial carotid cannulation was performed.

A small skin incision over interscapular BAT was made, exposing the caudal extreme of the iBAT. Connective tissue overlaying the caudal margin of the iBAT was separated, and thermocouple placed underneath the BAT was fixed (stitched or glued) in place. Back and belly thermocouples were then put in place (subcutaneously) and rectal thermocouple (core temperature) was inserted.

Following induction of anaesthesia, ICV treated rats were placed into a stereotaxic frame (SAS-4100; ASI Instruments, Warren, MI, USA) and ICV guide cannula were implanted using coordinates from a standard rat brain stereotaxic atlas^46^ (0.8 mm posterior, 1.5 mm lateral, and 3.5 mm ventrally from Bregma) to locate the lateral ventricle. An injection cannula was inserted to the guide cannula and injection of treatment was then made. To prevent back flow through the injection tract the injection cannula remained in place for 10 min following the injection. The guide cannula was then replaced.

### Experimental procedure

The study was conducted as a parallel study design. Standard procedures were adopted for measurement of outcome variables for all groups. Animals experienced food withdrawal on the day prior to the experiment. Water was available. To measure heart rate and blood pressure an intra-arterial carotid cannulation was performed. Anaesthetic depth was monitored and recorded throughout the experimental procedure including (a) monitoring respiratory rate, (b) spontaneous movement, (c) monitoring arterial blood pressure, (d) withdrawal reflex to toe pinch, and (e) eye blink reflex to light touching around the eye. Administration of urethane IV (1–1.5 mg/kg, Sigma-Aldrich) was administered at any sign of anaesthetic recovery.

Temperature was measured using a series of thermocouples placed underneath the iBAT depot, underneath the skin of the back, underneath the skin of the belly, and inserted rectally (wire type K thermocouple; JAYCAR, catalogue no. QM1283).

Throughout the experiment the rats were exogenously warmed using an electric heating blanket. Constant electrical warming was applied, and warming was maintained to ensure that core temperature did not fall below normal, prior to pharmacological intervention. Prior to pharmacological intervention, stable core and iBAT temperature was maintained for 30 min. The current applied did not change during the entire experimental procedure, so heating effect is entirely thermogenic and not a result of altered heating blanket activity.

Animals in the IV groups were administered IV caffeine (10 mg/kg/0.3 ml, BDH) or saline (vehicle alone, 0.3 ml). Animals in the ICV groups were dosed via ICV injection of caffeine (5–10 μg/100 nl) or saline (vehicle alone). Doses of caffeine both IV and ICV were based on previous studies measuring the stimulant, but non-anxiogenic doses caffeine, where the dose of caffeine did not promote any behavioural impairment^44–45^.

When the animal had recovered from the drug treatment (4 h post administration being the whole experiment), the animal then underwent whole body perfusion and the brain was extracted for immunohistochemistry analysis for c-Fos expression. The timing between the injection (ICV or IV; caffeine or vehicle alone) and the perfusion was consistent for all treatments.

### Perfusion and tissue processing

At the completion of the experimental procedure the animals were perfused using 10% neutral buffered (pH 7.4) formalin 4% formaldehyde solution. Brains were extracted and postfixed in the same fixative overnight. Tissue was immersed for 24 h in 30% sucrose in 0.1 M phosphate-buffered saline (PBS). Tissue processing and immunohistochemistry procedures were conducted as described elsewhere^59^. The antibodies which were used in the current study are described below.

### Antibodies for c-Fos immunohistochemistry

Immunohistochemistry was performed on every fourth coronal 30 µm section of the hypothalamic section, using a standard avidin–biotin–horseradish peroxidase complex (ABC) method, with a rabbit anti-Fos-polyclonal antibody (1:3000; catalogue no. ABE457;EMD Millipore Corp, USA), a biotinylated anti-rabbit IgG antibody (1:200; Vector Laboratories, Burlingame, CA, USA), ABC (1:200; Vectastain Elite ABC Kit; Vector Laboratories, Burlingame, CA, USA), and Vector SG (Peroxidase substrate kit; catalogue no. SK-4700; Vectastain Elite ABC Kit; Vector Laboratories, Burlingame, CA, USA) to produce a blue product.

### Cell counts—BLA, PaAP, MPA, LH, PeF/LH, DMH, VMH, CM and PVP

Two rostrocaudal sections (− 1.30 mm and − 1.40 mm from bregma) containing the basolateral amygdala (BLA), the paraventricular hypothalamic nucleus (PaAP), the medial preoptic area (MPA), and the LH of each rat were selected for analysis as shown in Fig. 3a,b. Three further rostrocaudal levels (− 3.14 mm, − 3.30 mm, and − 3.60 mm from bregma) of each rat were selected for analysis. These levels included the PeF/LH, the DMH, the ventromedial hypothalamus (VMH), the paraventricular thalamic nucleus posterior (PVP), and the central medial thalamus (CM), as shown in Fig. 4a–c. Round or oval shaped nuclei with black immunostaining darker than the background were counted as c-Fos-immunoreactive (c-Fos-ir). Darkfield and brightfield photomicrographs were taken with a 20× objective lens on an Olympus IX83 microscope with a Photometrics Prime 95B camera. Images were reconstructed via automatic stitching using CellSens software (Olympus). Darkfield photomicrographs were taken to reveal the neuroanatomy as shown in Fig. 3a–d, followed by brightfield photomicrographs taken of the same section without adjusting the stage^59^. Darkfield images were used to reveal the neuroanatomy, locate, and define the region of interest (ROI; Fig. 5c)^59^. Then definitions of the ROI were transferred to the brightfield image in preparation for automated cell-counting in ImageJ (Fig. 5d)^59^. Once the ROI’s were defined in each image regions were processed in ImageJ via the following commands: Image > Type > 8-bit; Image > Adjust > Threshold; Process > Binary > Watershed; Analyse > Analyse Particles (size: 40–100; Circularity: 0.80–1.00)^59^. Threshold could be moderately adjusted systematically to ensure enough contrast between cells and background. To ensure reliable results via the automated protocol a blinded assessor counted cells on 12 sections via the point tool in ImageJ.

### Statistical analysis

All statistical analysis of temperature data was conducted using IBM SPSS and PRISM 8 GraphPad software. Averages from the thermocouple (iBAT, and core), heart rate and mean arterial pressure data were calculated for 5-min intervals from baseline to 150 min post injection for both intervention groups. Changes from baseline were then calculated from the 5-min intervals. All data were inspected for normality, and where assumptions of normality were not met, data were transformed to meet assumptions of normality prior to statistical analysis. All data presented in the manuscript are raw data. Data for each outcome variable were analysed using separate two-way repeated measures ANOVA for each site of administration (ICV and IV). Each ANOVA assessed differences between treatments (caffeine, control) and time points. Where interaction effects were identified, differences between groups over time were assessed using Bonferroni post hoc pairwise comparisons.

For analysis of the c-Fos-ir, the automated counts of neurons immunolabelled for c-Fos were averaged for each ROI and animal. The ROI’s included BLA, PaAP, MPA, LH, PeF/LH, DMH, VMH, PVP, CM. The cell counts were compared between treated and control groups using separate two-way ANOVA for each site of administration (ICV and IV). Each ANOVA assessed the differences in c-Fos-ir counts between treatments and ROI’s. Where an interaction effect was identified, post hoc analysis using Bonferroni pairwise comparisons test to was used to determine which ROI’s were different between groups. Reliability of the automated cell count was assessed using intra-class correlation coefficient and reliability coefficients were interpreted as follows: below 0.50 poor, 0.50–0.75 moderate, and > 0.75 good^69^.
